# Eastern Equine Encephalitis Virus in Mexican Wolf Pups at Zoo, Michigan, USA

**DOI:** 10.3201/eid2704.202400

**Published:** 2021-04

**Authors:** Kimberly A. Thompson, Eileen Henderson, Scott D. Fitzgerald, Edward D. Walker, Matti Kiupel

**Affiliations:** Binder Park Zoo, Battle Creek, Michigan, USA (K.A. Thompson);; Michigan State University Veterinary Diagnostic Laboratory, Lansing, Michigan, USA (E. Henderson, S.D. Fitzgerald, M. Kiupel);; Michigan State University Department of Entomology, Lansing (E.D. Walker)

**Keywords:** Eastern equine encephalitis virus, meningoencephalitis, viruses, Michigan, mosquitoes, vector-borne infections, wolf, United States, meningitis/encephalitis

## Abstract

During the 2019 Eastern equine encephalitis virus (EEEV) outbreak in Michigan, two 2-month old Mexican wolf pups experienced neurologic signs, lymphohistiocytic neutrophilic meningoencephalitis with neuronal necrosis and neuronophagia, and acute death. We identified EEEV by reverse transcription real-time PCR and in situ hybridization. Vector mosquitoes were trapped at the zoo.

In North America, Eastern equine encephalitis virus (EEEV; family *Togaviridae*, genus *Alphavirus*) occurs as an enzootic cycle between mosquitoes (primarily *Culiseta melanura*) and passerine birds within freshwater hardwood swamps (*1*–*3*). When favorable ecologic conditions occur, EEEV prevalence increases via amplification until spillover transmission occurs into humans and equids, and less commonly other species (*1*). Other mosquito species, such as *Coquilettidia perturbans* and *Aedes vexans*, act as bridge vectors by preferentially feeding on mammals; they may be responsible for the epizootic cases among mammalian hosts (*2*). Outbreaks of EEEV in the northern United States occur intermittently between years but during a predictable time of the year, late summer through early fall (*3*).

Among the naturally occurring encephalitic alphaviruses, EEEV has the highest mortality rate in humans (50%–75%) and equids (70%–90%) (*4*). Additional reports exist of clinical disease in a wide variety of mammalian and avian species, including swine, cattle, white-tailed deer, alpacas, seals, domestic canids, pheasants, emus, penguins, and cassowary birds (*5*–*10*). Clinical signs of EEEV range from asymptomatic infection to severe and often fatal neurologic disease; signs may include pyrexia, anorexia, recumbency, diarrhea, ataxia, seizures, nystagmus, and head pressing. Previous reports of EEEV in domestic canids have been rare and have mainly been in young puppies <6 months of age (*9*,*10*). Serologic evidence suggests that exposure to EEEV in free-ranging gray wolves (*Canis lupus*) is low (0% of pups and 3% of adults) in Minnesota (*11*), where EEEV is uncommon.

## The Study

In 2019, the southwest region of Michigan experienced high incidence of EEEV exposure in humans and nonhuman animals, including wild deer and domestic horses (*7*,*8*,*12*). Clinical human cases were reported during June 18–September 20, 2019 (*12*). Binder Park Zoo (BPZ) (Battle Creek, Michigan, USA) is on a 400-acre property, half maintained as natural wetlands with a large population of native waterfowl and passerine avian species and half as a developed zoo. The Mexican wolf exhibit is located ≈30 m from the natural wetlands. In the midst of the outbreak, EEEV was diagnosed in two 2-month old Mexican wolf (*Canis lupus baileyi*) pups at BPZ. We assumed that transmission of EEEV to the wolf pups was from a mosquito bite that occurred at their exhibit location.

**Figure 1:**
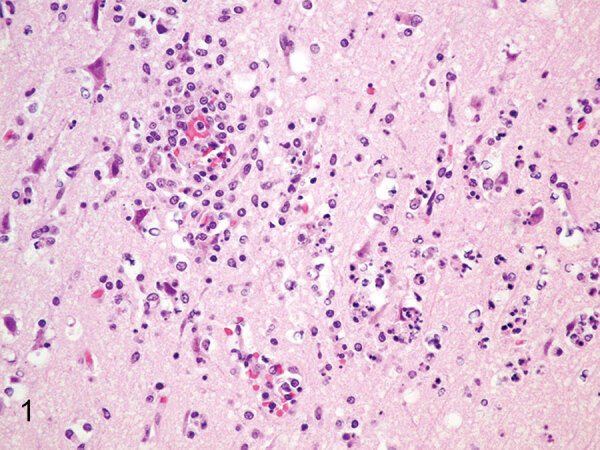
Brain specimen from Mexican wolf pup infected with eastern equine encephalitis virus at Binder Park Zoo, Michigan, USA. Hematoxylin and eosin stain shows severe, acute necrotizing and neutrophilic encephalitis with neuronal necrosis with pyknotic nuclei associated with perineuronal satellitosis and neutrophilic neuronophagia.

**Figure 2:**
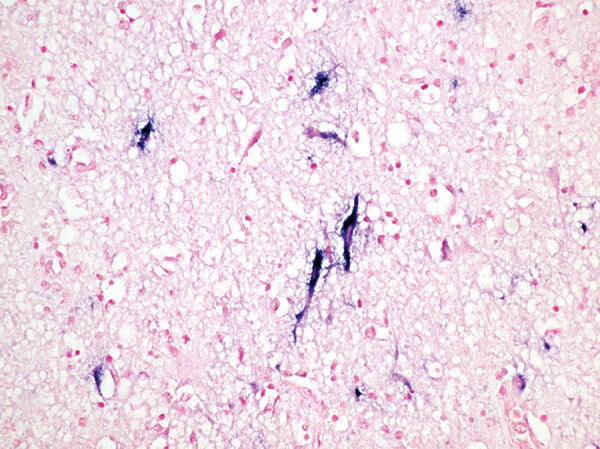
Brain specimen from Mexican wolf pup infected with eastern equine encephalitis virus (EEEV) at Binder Park Zoo, Michigan, USA. Blue stain shows EEEV nucleic acid in the perikaryon and dendrites of necrotic and intact neurons. Nuclear fast red counterstain shows nitro blue tetrazolium/5-Bromo-4-chloro-3-indolyl phosphate (NBT/BCIP) chromogen.

Pup 1 was a 2-month-old male pup that was brought for care on September 1, 2019, after a brief entanglement with the exhibit’s electric fence. The pup was noted to be increasingly ataxic, which progressed quickly to an obtunded recumbent state. On examination, the pup had decreased responsiveness to handling, increased respiratory effort and crackles, anisocoria, and pyrexia (temperature of 40°C; in adult domestic canids, pyrexia is >40°C) (*9*). Initial supportive care was unsuccessful; the pup died shortly thereafter. Necropsy revealed a focal area of hemorrhage along the junction of the basilar and posterior cerebral arteries. We submitted formalin-fixed tissues to Michigan State University Veterinary Diagnostic Laboratory (MSU VDL; Lansing, Michigan, USA) for histopathology. Throughout the cerebrum we observed expansion of the Virchow-Robin space by large numbers of lymphocytes and histocytes with extension of the inflammatory cells into the surrounding neuropil. Gray and white matter had randomly scattered foci of rarefaction and necrosis with low numbers of associated infiltrating neutrophils (Figure 1). Inflammatory cells often surrounded and occasionally phagocytize necrotic neurons in the process of neuronophagia. The meninges were expanded by edema and moderate numbers of lymphocytes and histiocytes. The cerebellum and brain stem had similar lesions. The cerebrum was positive for EEEV on SYBR green–based real-time reverse transcription PCR (rRT-PCR); EEEV nucleic acid was detected within neurons via in situ hybridization (Figure 2) (*13*,*14*). We noted a mild eosinophilic pneumonia.

Pup 2 was found deceased in the underground den the following day. This male pup had a history of healing traumatic rib fractures and delayed growth rate; he was in treatment for pneumonia and a suspected hepatic abscess and had been improving. Necropsy revealed healed rib fractures and consolidated left cranial lung lobes; the liver was firm, with a pronounced lobular pattern and prominent white interlobular septae. We sent formalin-fixed tissues for histopathology at MSU VDL. The lesions within the brain were similar to those described for pup 1. In addition, there was a moderate lymphoplasmacytic bronchointerstitial pneumonia and severe chronic fibrosing periportal hepatitis, moderate bile duct hyperplasia, moderate arteriole proliferation, and intermittent absence of periportal veins (portal vein hypoplasia), most consistent with a congenital vascular anomaly. Culture of the lung was positive for rare *Mycoplasma canis* and moderate *Escherichia coli* (negative for virulence factor genes *cnf1* and *cnf2*). The brain was positive by rRT-PCR for EEEV; in situ hybridization detected EEEV nucleic acid (*13*,*14*).

The surviving female pup (pup 3) and the dam and sire showed no clinical signs. Banked serum samples frozen at −30°C were sent to National Veterinary Services Laboratories (Ames, Iowa, USA) for plaque reduction neutralization test to evaluate timelines of exposure for pups 1, 2, and 3. Samples from pups 2 and 3 were negative, whereas serum from pup 1 tested positive for neutralizing antibodies at 1:10 dilution on the day of death (Table 1). Frozen cerebrum from the fourth littermate (pup 4) that had died a month earlier at 4 weeks of age tested negative by rRT-PCR for EEEV (*13*,*14*). Necropsy findings from pup 4 included thoracic rib fractures and moderate, acute, diffuse, fibrinosuppurative bacterial alveolitis and pleuritis.

**Table 1:** Plaque reduction neutralization test results for eastern equine encephalitis virus in banked serum samples from 3 Mexican wolf pups at Binder Park Zoo, Michigan, USA*

Animal ID	Date	Result
Pup 1	2019 Aug 14	Negative
	2019 Sep 1	Positive
Pup 2	2019 Aug 12	Negative
	2019 Aug 22	Negative
Pup 3	2019 Aug 14	Negative
	2019 Sep 20	Negative

*Test result interpreted at 1:10. Pups were tested before (all pups), during (pup 2), and after (pup 3 only) an outbreak of eastern equine encephalitis.

We conducted mosquito surveillance throughout the zoo’s property using dry ice–baited, CDC miniature light traps (John W. Hock Company, https://www.johnwhock.com) on 3 different dates (September 25 and 27, October 9). Mosquitoes were identified to species, and pools of <25 individuals were tested for EEEV RNA by rRT-PCR (*15*). Both the enzootic vector, *Culiseta melanura* (n = 6/378), and possible bridge vector mosquito species such as *Coquillettidia perturbans* (n = 15/378) were present (Table 2). After risk assessment, with local and state agencies, mosquito management was implemented on the property, including targeted barrier and state conducted adulticide spray over the area. All pools were negative for EEEV RNA.

**Table 2:** Results from 3 rounds of mosquito trapping at Binder Park Zoo, Michigan, USA*

Mosquito species	Trap date		
	2019 Sep 25	2019 Sep 27	2019 Oct 9
*Aedes cinereus*	1	2	0
*Aedes japonicus*	8	3	0
*Aedes trivittatus*	107	58	6
*Aedes vexans*	72	22	1
*Anopheles punctipennis*	3	1	0
*Anopheles quadrimaculatus*	11	9	1
*Anopheles walkeri*	1	0	0
*Coquillettidia perturbans*	8	7	0
*Culex erraticus*	0	7	1
*Culex pipiens*	4	2	0
*Culiseta melanura*	4	2	0
*Culex territans*	0	1	0
*Orthopodomyia signifera*	1	0	0
*Psorophora ferox*	21	5	0
*Uranotaenia sapphirina*	6	1	4
Total no. mosquitoes	247	118	13
Total species	13	13	5
Total no. traps	10	13	12
Overnight temperature, °C	16–22	19–21	11–18

*After detection of eastern equine encephalitis virus, mosquitoes were trapped with CDC miniature light traps baited with dry ice. Adulticide spray was applied between the second and third round. Data are numbers of mosquitoes except where otherwise noted.

## Conclusions

Zoonotic disease detection, especially for reportable diseases, may have implications to a zoo beyond animal health and may require a substantial amount of time and resources. After diagnosis of EEEV cases, the zoo provided educational material on EEEV and complimentary DEET mosquito spray for staff and zoo patrons. In addition, all overnight camping safaris, evening events, and school field trip groups were canceled. Zoos can act as sentinels for disease detection in an area because of the wide variety of resident species and thorough necropsies. Since the diagnosis of EEEV, BPZ’s preventative medicine measures to decrease the risk for nondomestic canids to contract EEEV include the use of a monthly topical pyrethrin-based product on all Mexican wolves starting at 8 weeks of age. 

Because of the natural distribution of the Mexican wolf population in Mexico and the southwestern United States, EEEV probably has little effect on the free-ranging population. However, the translocation of animals to zoos outside of their natural range may expose them to novel diseases such as EEEV. The cases in our study provide evidence for clinicians to include EEEV as a differential for acute neurologic signs or death in young canids, both domestic and nondomestic, especially during outbreaks of EEEV.
